# *Tn*P and AHR-CYP1A1 Signaling Crosstalk in an Injury-Induced Zebrafish Inflammation Model

**DOI:** 10.3390/ph17091155

**Published:** 2024-08-31

**Authors:** Geonildo Rodrigo Disner, Thales Alves de Melo Fernandes, Milton Yutaka Nishiyama-Jr, Carla Lima, Emma Wincent, Monica Lopes-Ferreira

**Affiliations:** 1 Immunoregulation Unit, Laboratory of Applied Toxinology (CeTICS/FAPESP), Butantan Institute, São Paulo 05585-000, Brazil; geonildo.disner.esib@esib.butantan.gov.br (G.R.D.); carla.lima@butantan.gov.br (C.L.); 2 Unit of System Toxicology, Institute of Environmental Medicine, Karolinska Institutet, 171 77 Solna, Sweden; emma.wincent@ki.se; 3 Nucleus of Bioinformatics and Computational Biology, Laboratory of Applied Toxinology, Butantan Institute, São Paulo 05585-000, Brazil; thales.fernandes.esib@esib.butantan.gov.br (T.A.d.M.F.); milton.nishiyama@butantan.gov.br (M.Y.N.-J.)

**Keywords:** cytochrome P450, drug discovery, inflammation, Tg(cyp1a:EGFP), toxinology, zebrafish

## Abstract

Aryl Hydrocarbon Receptor (AHR) signaling is crucial for regulating the biotransformation of xenobiotics and physiological processes like inflammation and immunity. Meanwhile, *Thalassophryne nattereri* Peptide (*Tn*P), a promising anti-inflammatory candidate from toadfish venom, demonstrates therapeutic effects through immunomodulation. However, its influence on AHR signaling remains unexplored. This study aimed to elucidate *Tn*P’s molecular mechanisms on the AHR–cytochrome P450, family 1 (CYP1) pathway upon injury-induced inflammation in wild-type (WT) and *Ahr2*-knockdown (KD) zebrafish larvae through transcriptomic analysis and *Cyp1a* reporters. *Tn*P, while unable to directly activate AHR, potentiated AHR activation by the high-affinity ligand 6-Formylindolo [3,2-b]carbazole (FICZ), implying a role as a CYP1A inhibitor, confirmed by in vitro studies. This interplay suggests *Tn*P’s ability to modulate the AHR-CYP1 complex, prompting investigations into its influence on biotransformation pathways and injury-induced inflammation. Here, the inflammation model alone resulted in a significant response on the transcriptome, with most differentially expressed genes (DEGs) being upregulated across the groups. *Ahr2*-KD resulted in an overall greater number of DEGs, as did treatment with the higher dose of *Tn*P in both WT and KD embryos. Genes related to oxidative stress and inflammatory response were the most apparent under inflamed conditions for both WT and KD groups, e.g., *Tnfrsf1a*, *Irf1b*, and *Mmp9*. *Tn*P, specifically, induces the expression of *Hspa5*, *Hsp90aa1.2*, *Cxcr3.3*, and *Mpeg1.2*. Overall, this study suggests an interplay between *Tn*P and the AHR-CYP1 pathway, stressing the inflammatory modulation through AHR-dependent mechanisms. Altogether, these results may offer new avenues in novel therapeutic strategies, such as based on natural bioactive molecules, harnessing AHR modulation for targeted and sustained drug effects in inflammatory conditions.

## 1. Introduction

Animal venoms are a rich source of natural proteins and peptides that can modulate the activity of a wide range of cellular interactions, e.g., neuronal ion channels and receptors [1,2]. Toxin-isolated peptides can be chemically defined as molecular tools to discover new targets prone to trigger biological responses of great interest for human therapy. The novel cyclic *Thalassophryne nattereri* Peptide (*Tn*P) is an anti-inflammatory drug candidate isolated from the venom of the Brazilian venomous toadfish *Thalassophryne nattereri* Steindachner, 1876 [3]. This peptide is a promising therapeutic agent since it has demonstrated anti-inflammatory and anti-allergic properties in preclinical analysis. These studies support its therapeutic use and efficacy to control neuroinflammation and prevent demyelination in diseases such as multiple sclerosis [4], as well as for the treatment of asthma in murine models [5].

Cytochrome P450 (CYP) is an evolutionary conserved superfamily of enzymes that catalyzes the biotransformation of endogenous and exogenous chemicals in the body. Understanding their regulation and functions is essential for assessing the consequences of drug–drug interactions (DDIs) and metabolism. Several CYP genes, particularly those of the CYP1 family (*Cyp1a1*, *Cyp1a2*, and *Cyp1b1*) are upregulated due to the activation of the Aryl Hydrocarbon Receptor (AHR) [6]. The AHR is a transcription factor and environmental sensor that is activated upon binding to specific endogenous or exogenous receptor ligands, resulting in the upregulation of genes harboring a corresponding response element in their promoter region [7]. Besides the CYP1 enzymes, AHR may additionally regulate the expression of a large battery of genes, whose functions are related to processes such as inflammation, stem cell differentiation, cell cycle regulation, cancer stem cell expansion, and lipid accumulation [8,9,10,11,12,13].

The AHR-CYP1 constitutes a negative feedback mechanism that involves the AHR-dependent transcriptional induction of CYP1 genes, leading to increased expression of the corresponding enzymes. These enzymes then metabolize the AHR-activating ligands, promoting their degradation or conversion into less toxic forms. As a result, the levels of activating ligands decrease, reducing further AHR signaling and subsequent expression of CYP1 enzymes. This synchronized crosstalk is pivotal in maintaining physiological homeostasis and mediating toxicity effects by regulating the response to environmental and endogenous stimuli [14]. It has been demonstrated that the *Cyp1a1* gene is autoregulated by the action of its product protein and that this feedback mechanism is essential for the appropriate timing, duration, and magnitude of normal cellular functions regulated by AHR. In addition, inhibition of CYP1 prolongs the induction of AHR activity, disturbing the feedback regulation of ligand levels [15].

The zebrafish (*Danio rerio*) provides valuable contributions as an experimental model in drug discovery and toxicology, primarily when used in the embryo–larval stage since it is compatible with the 3Rs paradigm of animal experimentation. This is further strengthened by its high genetic homology with humans, conserved vertebrate biology, high fecundity, rapid development, and transparency at early life stages, empowering the zebrafish as an alternative to mammalian models [16].

Zebrafish can also be a powerful tool for the study of innate immunity and inflammation due to the conserved biological pathways and inflammatory responses, which can be assessed through commonly applied inflammation models such as by lipopolysaccharide (LPS) or copper sulfate (CuSO_4_) challenge and tail fin amputation [17]. Although zebrafish harbor three AHR genes (*Ahr1a*, *Ahr1b*, and *Ahr2*) compared to mammalians that only express one, zebrafish is a commonly used model to study AHR-mediated functions and chemical toxicity [18]. Among the zebrafish AHR copies, *Ahr2* is the most extensively studied, particularly for its roles in toxic responses, xenobiotic metabolism, immune response, and development [19].

We hypothesize that *Tn*P modulates AHR signaling, leading to the repression of inflammatory genes to restore immune homeostasis. In that context, this study aims to elucidate the putative interplay between *Tn*P and the AHR-CYP1 pathway and the molecular mechanism(s) underlying the impact of *Tn*P on AHR function upon injury-induced inflammation in zebrafish larvae.

## 2. Results and Discussion

### 2.1. TnP Disrupts the AHR-CYP Signaling Pathway

The AHR acts as a sensor of a broad range of chemicals and small molecules, which led to its recognition as a key player in the body’s defense against harmful compounds and health homeostasis through the activation of enzymes responsible for chemical transformation and metabolism [20,21]. Considering such vital contributions, the main featured enzymes related to the AHR are the cytochrome P450 1 (CYP1) enzymes due to their role in the metabolism of AHR ligands and pollutants. Thus, the AHR-CYP1 axis gained a significant focus in various research fields, where it was shown to influence the differentiation of immune cells and cytokine production, for example, impacting immune system functions [8,22,23]. With this, the AHR field transitioned from environmental toxicology to medical sciences, with a considerable focus on immune system regulation. This shift has since then expanded the knowledge of critical physiological functions of the AHR, reflecting its multifaceted roles in physiology and toxicology.

Several enzymes belonging to the CYP superfamily such as CYPs 1A1, 1A2, 2C9, 2C19, 2D6, and 3A4 have conventionally accounted for ~90% of CYP reactions with drugs [24,25]. However, the structure–metabolism relationship of CYP substrates is complex and needs further investigation [25]. Zebrafish express 94 CYP genes distributed among 18 gene families [26], including five CYP1 genes: *Cyp1a*, *Cyp1b1*, *Cyp1c1*, *Cyp1c2*, inducible by AHR agonists, and *Cyp1d1*, a constitutively expressed *Cyp1a-*like gene [27].

**Figure 1:**
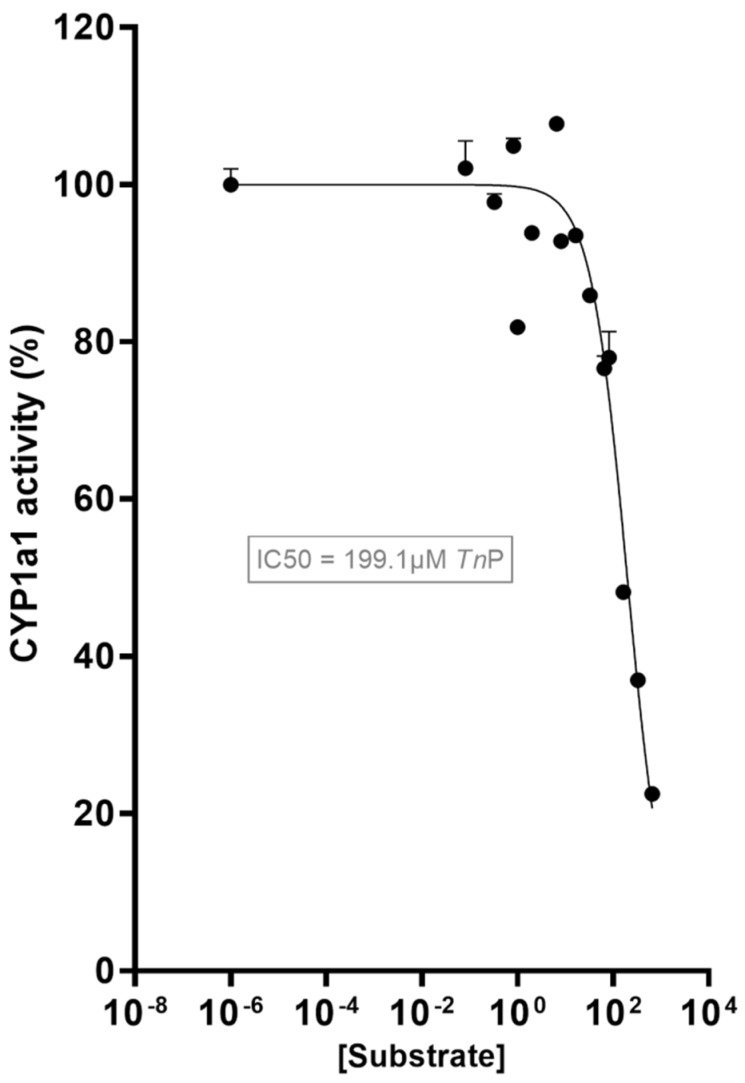
*Tn*P suppresses CYP1A1 activity in vitro. The effect of *Tn*P (82.5 nM–660 µM) on the enzyme activity revealed the 50% inhibitory concentration (IC50) as 199.1 µM. Analysis of log(inhibitor) vs. response—variable slope performed by GraphPad Prism Software (San Diego, CA, USA).

In this study, we investigated the impact of the candidate peptide *Tn*P with the AHR-CYP1 pathway, hypothesizing that a small molecule such as *Tn*P may interact with this pathway. Interestingly, *Tn*P exhibited an inhibitory effect on CYP1A1 activity as determined using a recombinant CYP1A1 enzyme in vitro (Figure 1; Supplementary Table S1). Through a range of exposures (0.0825–660 μM), the *Tn*P concentration causing 50% inhibition of activity (IC50) was determined to be 199.1 μM.

**Figure 2:**
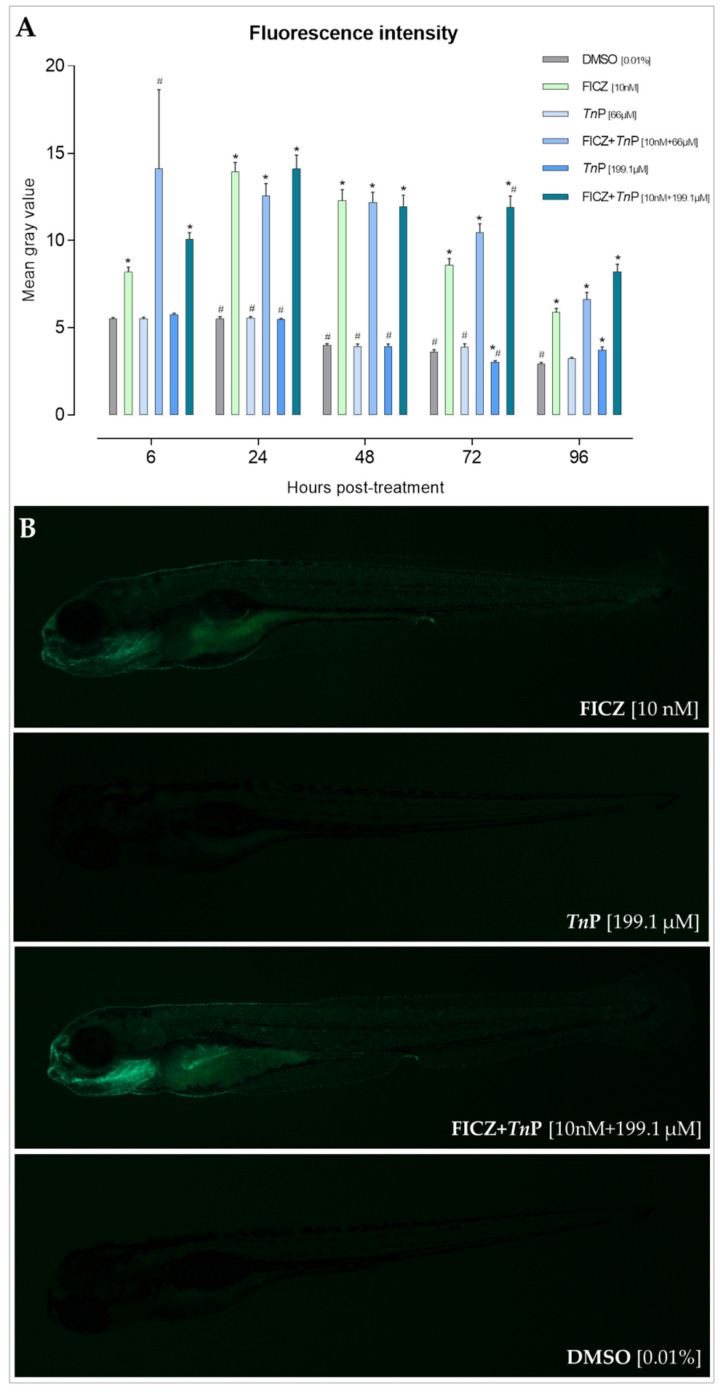
*Tn*P sustains *Cyp1a* expression induced by FICZ. (**A**) Fluorescence intensity analysis measured by ImageJ of Tg(*cyp1a*:EGFP) zebrafish reporter embryos treated with FICZ and/or *Tn*P to assess *cyp1a* expression over time, with exposure to the compounds starting at 24 h post-fertilization (hpf). DMSO 0.01% *v*/*v* was used as a negative control. Graph produced using GraphPad Prism Software (San Diego, CA, USA). * represents a significant difference (*p* < 0.05) to the negative control (DMSO), and # represents a significant difference (*p* < 0.05) to FICZ. (**B**) Selected representations of Tg(*cyp1a*:EGFP) zebrafish reporter embryos to assess the intensity and spatial distribution of AHR activation through *Cyp1a* expression at 96 h post-treatment (hpt). Abbreviations: FICZ—6-Formylindolo[3,2-b]carbazole; *Tn*P—*Thalassophryne nattereri* Peptide; DMSO—Dimethyl Sulfoxide.

To connect the inhibition with effects on the AHR-CYP1 axis in vivo, we next determined the AHR-activating potency of *Tn*P using a transgenic zebrafish line that reports AHR activation, by the induced expression of *cyp1a*:EGFP. Tg(*cyp1a*:EGFP) reporter embryos were exposed to the high-affinity AHR ligand 6-Formylindolo[3,2-b]carbazole (FICZ, 10 nM) and the *Tn*P (66 and 199.1 µM (IC50)), separately or together, starting at 1 day post-fertilization (dpf) (Figure 2A). The *cyp1a:EGFP* expression was monitored daily until 96 h post-treatment (hpt) through fluorescence imaging (Figure 2B).

In the reporter experiment, *Tn*P alone did not indicate any AHR activation. However, as notably evidenced from 72 hpt onward, in co-exposure with FICZ, *Tn*P sustained a greater *Cyp1a* induction. This observation corroborates its property as a CYP1A1 enzyme inhibitor, as FICZ clearance is dependent on CYP1, which if inhibited will extend the AHR activating capacity by FICZ [15,28]. Hence, though *Tn*P is not a direct AHR ligand, this is the first evidence showing its ability to indirectly activate AHR by hampering the metabolic clearance of the receptor ligands.

**Figure 3:**
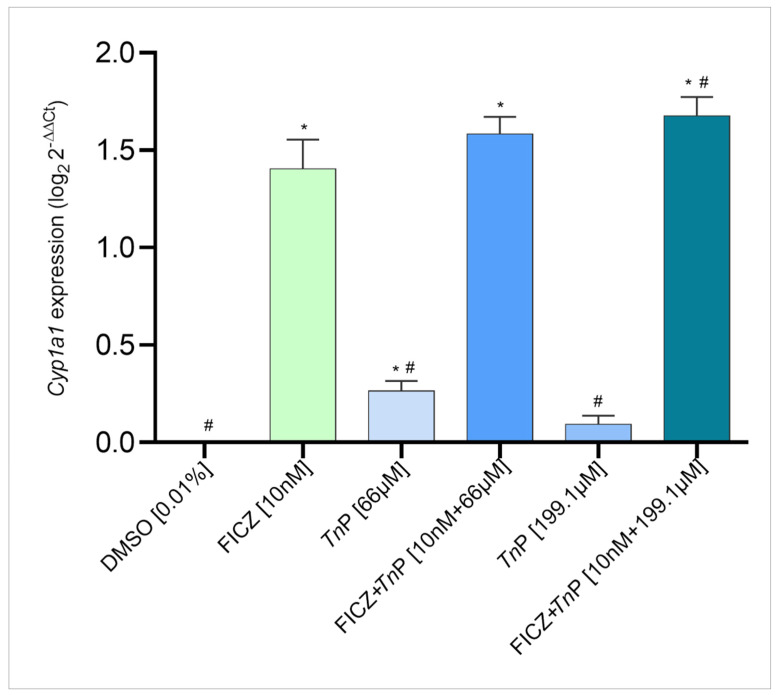
*Tn*P enhances *Cyp1a1* expression in zebrafish embryos. The expression level was assessed by qPCR at 96 h post-treatment, where embryos were treated with 10 nM 6-Formylindolo[3,2-b]carbazole (FICZ) and/or *Tn*P (66 and 199.1 µM). Exposure to chemicals starting at 24 h post-fertilization (hpf). DMSO 0.01% *v*/*v* was used as negative control (NC). * *p* < 0.05 with NC (DMSO); # *p* < 0.05 with positive control (10 nM FICZ). Produced using GraphPad Prism Software (San Diego, CA, USA).

To confirm these observations, *Cyp1a1* gene expression levels were determined by real-time PCR (qPCR) in the same Tg(*cyp1a*:EGFP) zebrafish embryos at 96 hpt. As seen in Figure 3, in the presence of 199.1 µM *Tn*P, the FICZ-induced AHR activation is enhanced.

While the role of CYP1 enzymes in biotransforming xenobiotics is well known [12], excessive CYP1A activity may also cause the formation of reactive oxygen species (ROS) [29]. For instance, the AHR-dependent induction of CYP1 is the primary source of ROS in hepatocytes incubated with 2,3,7,8-Tetrachlorodibenzo-p-dioxin (TCDD) or exposed to polycyclic aromatic hydrocarbons (PAHs) such as benzo[a]pyrene (BaP), as reviewed by Rothhammer and Quintana [30]. CYP1-mediated overproduction of ROS may in turn affect cell metabolism and proliferation, owing to the direct and indirect activation of several signaling pathways including NF-κB or oncoproteins c-Jun or Rb [31,32].

Besides CYP1, several enzymatic systems, including NADPH oxidase (NOX), cyclooxygenase (COX), and possibly aldo-keto reductase (AKR) 1, are regulated through the AHR signaling pathway in terms of their ability to generate ROS in various cell types and tissues [33].

Although speculative, *Tn*P’s potential to inhibit CYP1A1 may add to its therapeutic effect by reducing levels of ROS. Our data also suggest that *Tn*P may affect the metabolism of other drugs and could thereby be beneficial or adverse in combination with other medicines relying on CYP metabolism for their clearance or bioactivation.

Recent studies on the association between *Cyp1a1* levels and disease further stress the importance of a tightly controlled regulation of this gene, as constitutive expression was shown to exacerbate immune cell activation and skin pathology [34] and impaired intestinal immune function and stem cell differentiation [35,36] in different mouse models. In this regard, *Tn*P, through its putative inhibitory role, could help alleviate inflammatory conditions resulting from exacerbated CYP1 function.

In addition to its implication in the resolution of inflammation and tissue injury, Asnani et al. [37] recently concluded that inhibition of CYP1 was protective of (DOX)-induced cardiotoxicity. While this study, performed in zebrafish, focused on the mechanisms of visnagin-mediated cardioprotection, a later study by Lam et al. [38] screened 120 potential molecules that prevent DOX-induced cardiotoxicity, of which seven exhibited inhibitory activity towards CYP1.

While the addressed studies support a beneficial role of CYP1 inhibition in a clinical setting, this may also cause detrimental effects, stressing the need for an exhaustive DDI analysis to determine its effectiveness and safety in various contexts or combinations with other chemicals.

### 2.2. The Role of AHR in the Therapeutic Effect of TnP

**Figure 4:**
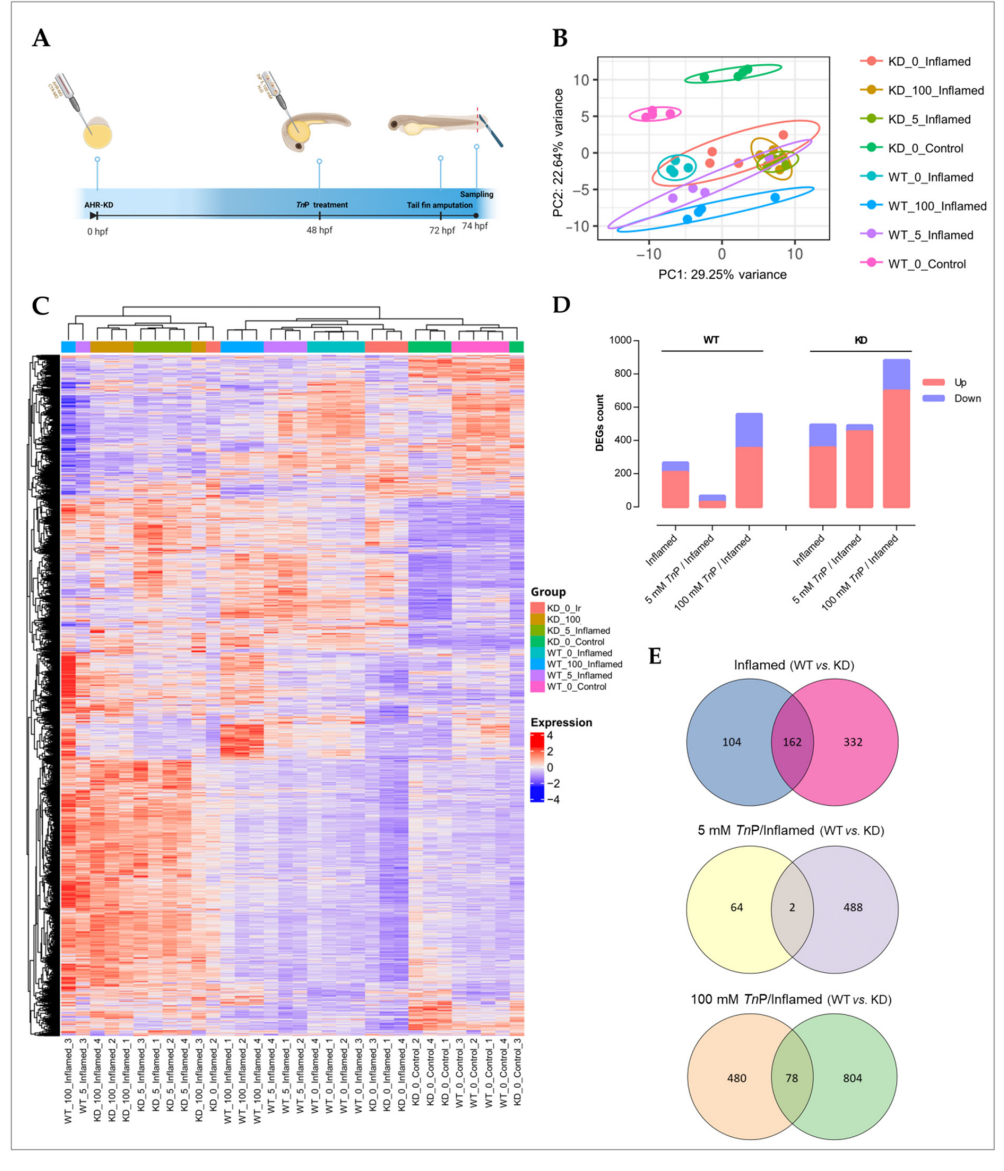
Transcriptomic analysis in a zebrafish inflammation model treated with *Tn*P. (**A**) Experimental design comprising *Ahr2*-knockdown (KD), *Tn*P prophylactic treatment, and injury-induced inflammation model by tail fin amputation. (**B**) Principal component analysis (PCA) for the overall distribution of the datasets; the ellipses limit the 95% confidence interval among biological replicates. (**C**) Hierarchical clustering heatmap depicting gene transcription patterns; adjust *p*-value < 0.05 and log_2_ fold change ±0.5. (**D**) Bar plots illustrating the differentially expressed genes (DEGs) between pairwise comparisons in both genotypes. (**E**) Venn diagrams of the overlapping and distinct DEGs in KD and wild-type (WT) groups modulated by *Tn*P under inflammatory conditions.

Knowing that *Tn*P affects AHR-CYP1 signaling, we subsequently investigated how *Tn*P acts in the context of injury-induced inflammation (hereafter referred to as inflamed) both in the presence and absence of *Ahr2* (Figure 4A). For this purpose, we performed a transcriptomic analysis of embryos with and without morpholino-mediated knockdown of *Ahr2*, under three scenarios, i.e., 0 mM *Tn*P/inflamed, 5 mM *Tn*P/inflamed, and 100 mM *Tn*P/inflamed. The resulting differentially expressed genes (DEGs) thereby demonstrate genes whose expression is modulated under inflammatory conditions, how these are associated with *Ahr2* function, and how *Tn*P affects these through *Ahr2*-dependent and -independent mechanisms. As seen in Figure 4B,C, the different experimental groups showed distinct effects on the transcriptome, illustrated by their clustering in the Principal Component Analysis (PCA) and hierarchical clustering. Notably, the controls that underwent inflammation only showed a strong separation from non-inflamed, as well as from the combination of inflamed and treatment, indicating that the inflammation itself constitutes a major source of transcriptional effects.

The number of DEGs in the different exposure scenarios clearly shows that the inflammation model based on tail fin amputation resulted in a noticeable response, with most DEGs upregulated across the groups (Figure 4D; Supplementary Figure S3). Knocking down *Ahr2* provoked a generally greater number of DEGs, as did the higher dose of *Tn*P in both wild-type (WT) and *Ahr2*-knockdown (KD) embryos. WT and KD presented similar trends except for the WT-5 mM *Tn*P/inflamed, which showed a reduced number of DEGs compared to WT-inflamed, while no difference was seen at a KD background. The distinct and overlapping DEGs from WT versus KD groups are presented in Figure 4E and in the supplement (Supplementary Figure S4 and Table S2).

Together, these results show the ability of *Tn*P treatment to modulate the number of DEGs, especially in the highest dose. The subtle effect at the lowest dose, significantly altering the expression of a small number of DEGs in WT larvae, contrasts with the *Ahr2*-KD group suggesting the role of this receptor in the anti-inflammatory effect of *Tn*P.

**Figure 5:**
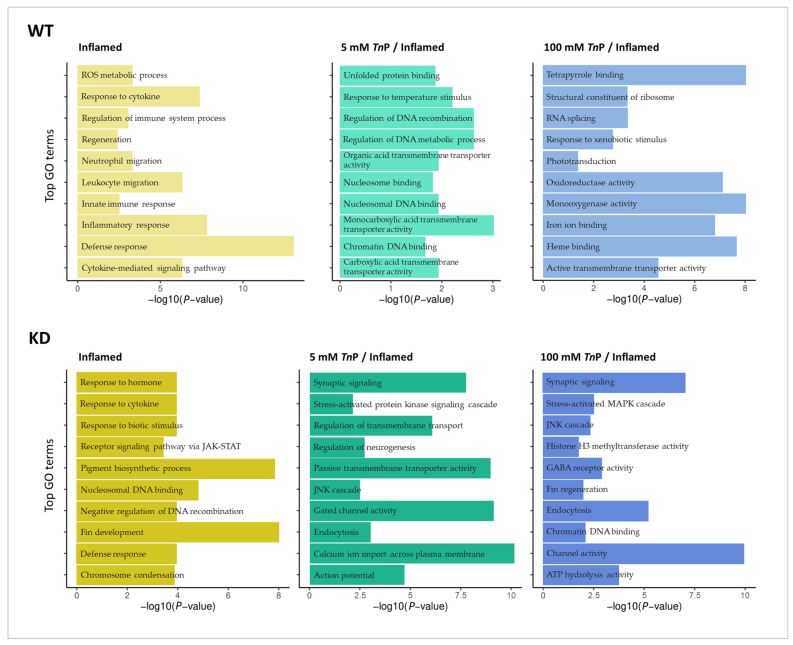
Effect of *Tn*P in a zebrafish inflammation model in wild-type (WT) or *Ahr2*-knockdown (KD) embryos observed through Gene Ontology (GO) enrichment analysis. The bar plots illustrate top enriched biological processes and molecular functions for differentially expressed genes between the following pairwise comparisons: inflamed vs. control (inflamed); treatment with *Tn*P 5/inflamed vs. inflamed (5 mM *Tn*P/inflamed); and treatment with *Tn*P 100/inflamed vs. inflamed (100 mM *Tn*P/inflamed).

Next, we used the DEG lists to perform a Gene Ontology (GO) enrichment analysis to view the biological processes and molecular functions most affected by the *Tn*P treatment in inflammation (Figure 5; Supplementary Table S3). In WT-inflamed, there were 84 enriched terms, which are related mainly to an acute inflammatory response, such as the cytokine-mediated signaling pathway, leukocyte and neutrophil migration, ROS metabolic process, and regeneration. *Tn*P treatment altered the molecular response to the inflammation, as observed by the enrichment analysis. Here, 5 mM *Tn*P resulted in 30 enriched GO terms, while 100 mM *Tn*P resulted in 128 enriched terms, including protein and nucleosome binding, tetrapyrrole binding, response to xenobiotic stimulus, oxidoreductase and monooxygenase activity, transmembrane transporter activity, regulation of DNA metabolic process, and recombination, among others (Figure 5).

Likewise, the GO enrichment analysis was performed with the *Ahr2*-KD groups, and the top terms are shown in Figure 5. The inflammatory response alone (KD-inflamed) resulted in 131 GO terms, many of which were not enriched in the WT-inflamed counterpart, such as pigment biosynthetic process and fin development. Upon treatment with 5 mM *Tn*P, 143 terms were enriched including endocytosis, synaptic signaling, and membrane channel transport, and 100 mM *Tn*P resulted in 218 enriched terms, with endocytosis, channel activity, stress-activated MAPK cascade, and JNK cascade being the top ones.

These results confirm that the inflammation induced by tail fin injury is regulated by the *Tn*P treatment. In addition to altering processes related to acute neutrophilic infiltration, it also induces biological responses responsible for membrane transport and the activity of enzymes from the heme-containing monooxygenases and oxidoreductase family, which catalyze a diverse range of oxidative reactions. Moreover, the absence of the *Ahr2* gene alters the *Tn*P effect, redirecting it towards pathways that regulate calcium transport through voltage-gated calcium channels (VGCCs) and GABA receptors. This shift has been substantiated by the enrichment of GO terms. There is evidence that certain types of VGCCs are targets for treating not only neuropathic but also inflammatory pain by GABA receptor agonists [39]. GABA and GABA type A receptor agonists decrease cytotoxic immune responses and cutaneous delayed-type hypersensitivity reactions [40].

**Figure 6:**
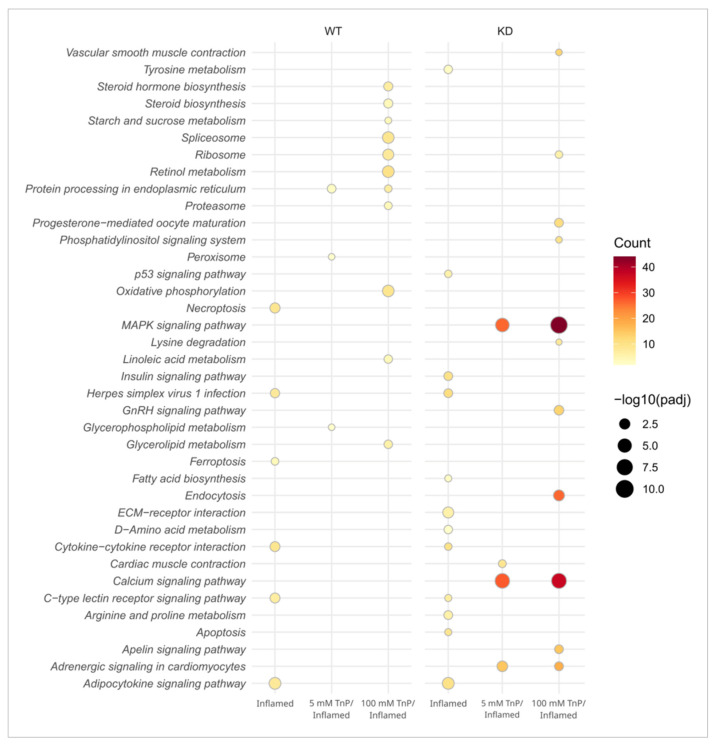
Effect of *Tn*P in a zebrafish inflammation model in wild-type (WT) or *Ahr2*-knockdown (KD) embryos observed through Kyoto Encyclopedia of Genes and Genomes (KEGG) enrichment analysis. The dot plot presents the list of enriched KEGG pathways based on differentially expressed genes between the following pairwise comparisons: inflamed vs. control (inflamed); treatment with *Tn*P 5/inflamed vs. inflamed (5 mM *Tn*P/inflamed); and treatment with *Tn*P 100/inflamed vs. inflamed (100 mM *Tn*P/inflamed) for both genotypes, i.e., wild-type (WT) and *Ahr2*-knockdown (KD).

Additionally, we performed a Kyoto Encyclopedia of Genes and Genomes (KEGG) enrichment analysis to expand the insights on affected pathways (Figure 6). KEGG is a knowledge database for systematically analyzing gene functions, linking genomics with higher functional information, and increasing the comprehension of functions of biological systems from molecular-level inputs produced by genome sequencing.

Here, we identified four enriched pathways under the inflammatory condition (inflamed) overlapping between WT and *Ahr2*-KD, i.e., cytokine–cytokine receptor interaction (dre04060), Adipocytokine signaling pathway (dre04920), C-type lectin receptor signaling pathway (dre04625), and Herpes simplex virus 1 infection (dre05168). These represent prototypical and crucial intercellular regulatory pathways engaged in innate and adaptive inflammatory host defenses. Also, they are canonically implicated in response to chemical or biological stimuli, cell growth, differentiation, cell death, angiogenesis, development, and repair processes aimed at restoring homeostasis. Apart from these four pathways, WT-inflamed and KD-inflamed exhibited several distinct enriched pathways, with all KD groups showing a greater number than their WT counterparts. Notably, in inflamed WT larvae, when treated with *Tn*P, we observed the intensification of pathways related to metabolism (e.g., steroid hormone biosynthesis, spliceosome, ribosome, and retinol metabolism) and oxidative phosphorylation.

Interestingly, mitogen-activated protein kinase (MAPK) signaling pathway (dre04010), calcium signaling pathway (dre04020), and endocytosis (dre04144) were the pathways more significantly enriched in KD groups treated with *Tn*P. Notably, the MAPK cascade is a highly conserved module involved in various cellular functions, including cell proliferation, differentiation, and migration. Being an important signaling pathway in inflammation, the observed stimulation by *Tn*P treatment under *Ahr2*-KD conditions provides insights into its anti-inflammatory potential related to *Ahr2* function. Additionally, calcium signaling is critical for cell communication and for driving intracellular processes, besides its relation to cardiac muscle contraction, suggesting a wider range of *Tn*P effects dependent on ARH.

**Figure 7:**
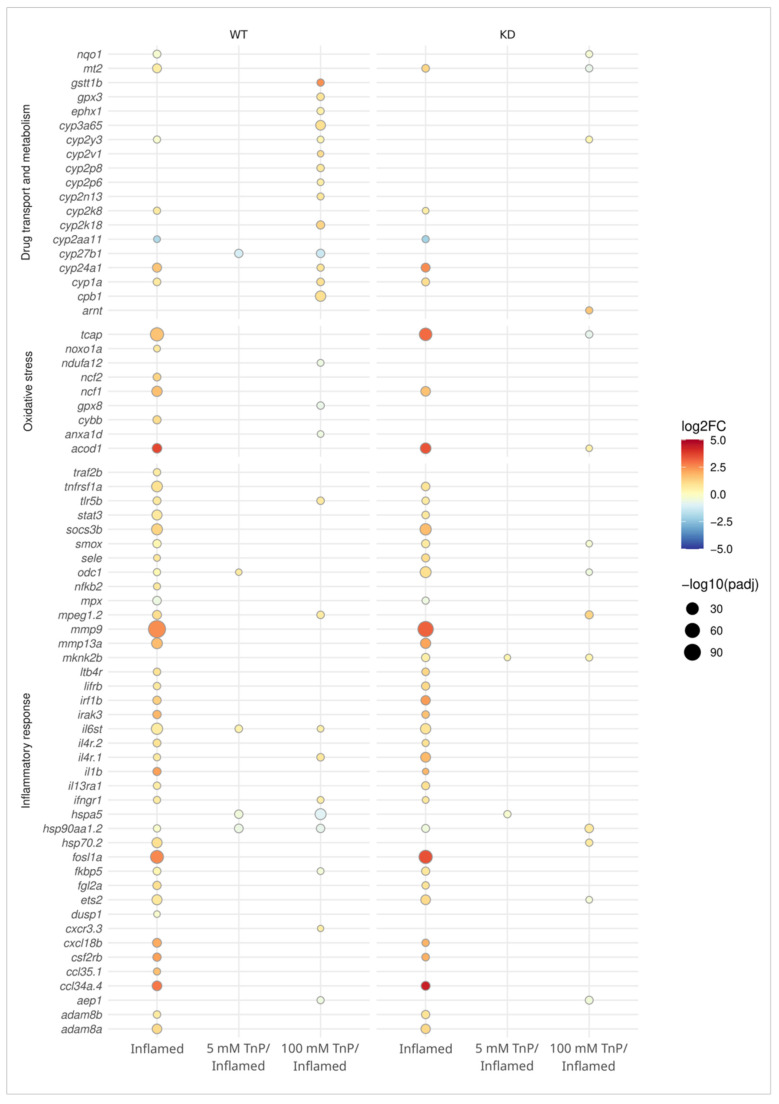
Dot plot illustrating the effect of *Tn*P treatment and inflammation in the expression profile of specific genes, in wild-type (WT) and *Ahr2*-knockdown (KD) embryos. These genes are known to regulate drug transport and metabolism (phase I and II), oxidative stress, and inflammatory response in the pairwise comparisons: inflamed vs. control (inflamed); treatment with *Tn*P 5/inflamed vs. inflamed (5 mM *Tn*P/inflamed); and treatment with *Tn*P 100/inflamed vs. inflamed (100 mM *Tn*P/inflamed) obtained from the RNAseq dataset. The dots represent statistically significant differences (adjust *p*-value < 0.05, log_2_FC ± 0.5), and their absence means no difference for that gene expression in the due comparison.

Based on the most statistically significant DEGs from our comparisons and those that repeatedly composed the main enriched GO terms, we selected a list of genes organized into three classes relevant to the questions addressed in this study, namely drug transport and metabolism, oxidative stress, and inflammatory response. The expression pattern of genes related to these classes is shown in Figure 7, illustrating their profile under the six comparative scenarios discussed herein, i.e., genes whose expression is modulated under inflammatory conditions and the effects on their expression under the *Tn*P treatment in WT and *Ahr2*-KD.

Notably, inflammation itself was able to activate AHR signaling, which corroborates its role in the acute inflammatory response. This was seen in WT-inflamed by the upregulation of *Cyp1a* and *Nqo1*. Likewise, in KD-inflamed, the *Cyp1a* was also upregulated even upon *Ahr2* knockdown, supporting the outcome of inflammation. However, a strong reduction in *Cyp1a* levels was seen when comparing *Ahr2*-KD and WT untreated controls (not inflamed), confirming the efficiency and reliability of the knockdown. Among other featured genes in the drug transport and metabolism category, many other CYP members were differentially expressed in both WT and *Ahr2*-KD upon inflammation. Notably, treatment of WT embryos with 100 mM *Tn*P resulted in the upregulation of several additional CYP genes, including *Cyp3a65*, *Cyp2k18*, *Cyp2p8*, *Cyp2p6*, and *Cyp2n13*, while *Cyp27b1* was downregulated. However, most of these genes were not differentially expressed after *Tn*P treatment in the KD group, suggesting that the presence of AHR may have a critical role in their regulation by *Tn*P (Figure 7).

As expected, the genes related to oxidative stress and inflammatory response were the most apparent under inflamed conditions for both WT and KD groups, albeit slightly fewer in the latter. Also, the expression level (log_2_FC) was notably higher for some of these in the KD group, e.g., *Tcap*, *Mmp9*, *Irf1b*, *Fosl1a*, and *Ccl34a.4* (Figure 7). Upon *Tn*P treatment, some of the genes involved in oxidative stress response showed a contrasting profile. The NADH:ubiquinone oxidoreductase subunit A12 (*Ndufa12*), glutathione peroxidase 8 (*Gpx8*), and annexin A1d (*Anxa1d*) had their expression downregulated when treated with 100 mM *Tn*P in WT but were not differentially expressed in KD. These genes are involved in the response to oxidative stress by acting on the mitochondrial respiratory chain or performing peroxidase activity, calcium ion binding activity, or phospholipase A2 inhibitor activity, indicating a role of the AHR in *Tn*P-mediated effects on those processes.

Early inflammatory response mainly involves the production of mediators required in the recruitment of immune cells to the site of damage. In that regard, both WT and *Ahr2*-KD displayed a functioning inflammatory response, for example, by increased expression of tumor necrosis factor receptor superfamily, member 1a (*Tnfrsf1a*), involved in neutrophil chemotaxis; interferon regulatory factor 1b (*Irf1b*), functional in the defense response to infection; matrix metallopeptidase 9 (*Mmp9*), involved in both extracellular matrix remodeling and in the inflammatory response by regulating the pro-inflammatory cytokines; and matrix metallopeptidase 13a (*Mmp13a*), involved in macrophage chemotaxis.

Additional genes upregulated under inflammation that cooperate with chemokine binding and activity are interleukin 6 cytokine family signal transducer (*Il6st*), interleukin 4 receptor, tandem duplicate 1 and 2 (*Il4r.1* and *Il4r.2*), chemokine (C-X-C motif) receptor 3, tandem duplicate 3 and ligand 18b (*Cxcl3.3* and *Cxcl18b*), chemokine (C-C motif) ligand 35, duplicate 1, and ligand 34a, duplicate 4 (*Ccl35.1* and *Ccl34a.4*). Interestingly, some of these were differentially expressed after *Tn*P treatment only in the presence of AHR (i.e., *Il6st*, *Il4r.1*, and *Cxcl3.3*).

In addition, prophylactic treatment with *Tn*P led to AHR-dependent reduction in the expression of the oxidative stress response genes *Ndufa12*, glutathione peroxidase 8 (*Gpx8*), and annexin A1d (*Anxa1d*), as well as AHR-dependent increase in drug transport and metabolism genes like glutathione S-transferase theta 1b (*Gstt1b*), glutathione peroxidase 3 (*Gpx3*), and epoxide hydrolase 1 (*Ephx1*).

Overall, within the genes involved in the inflammatory response, *Hspa5* had a different expression pattern when treated with *Tn*P (in both *Tn*P doses for WT and the lower dose for KD). When the expression of mediators involved in the inflammatory response is altered, it may have significant implications for the overall inflammatory process. Understanding the function of the specific genes involved, the nature of their differential expression, and the context in which these changes occur is crucial for unraveling the molecular mechanisms underlying inflammatory disorders and developing targeted therapeutic interventions. Research in this area is ongoing, and advancements in genomics and molecular biology continue to improve our understanding of the intricate regulatory networks governing the inflammatory response. Based on the expression profiles obtained in this study, it can be concluded that the AHR seems to have an elemental role in the inflammatory response regulation in the zebrafish model and that *Tn*P may exert at least part of its effects through AHR. Although the mechanism underlying this interaction is however not concluded yet, we can propose that the *Tn*P therapeutic effect via the AHR-CYP1 axis, inhibiting the inflammatory response, involves the participation of metabolic enzymes from the cytochrome P450 family and functions related to voltage-gated calcium channels.

### 2.3. Comparisons between Zebrafish and Human CYP1A1 and TnP-CYP1A1 Docking

**Figure 8:**
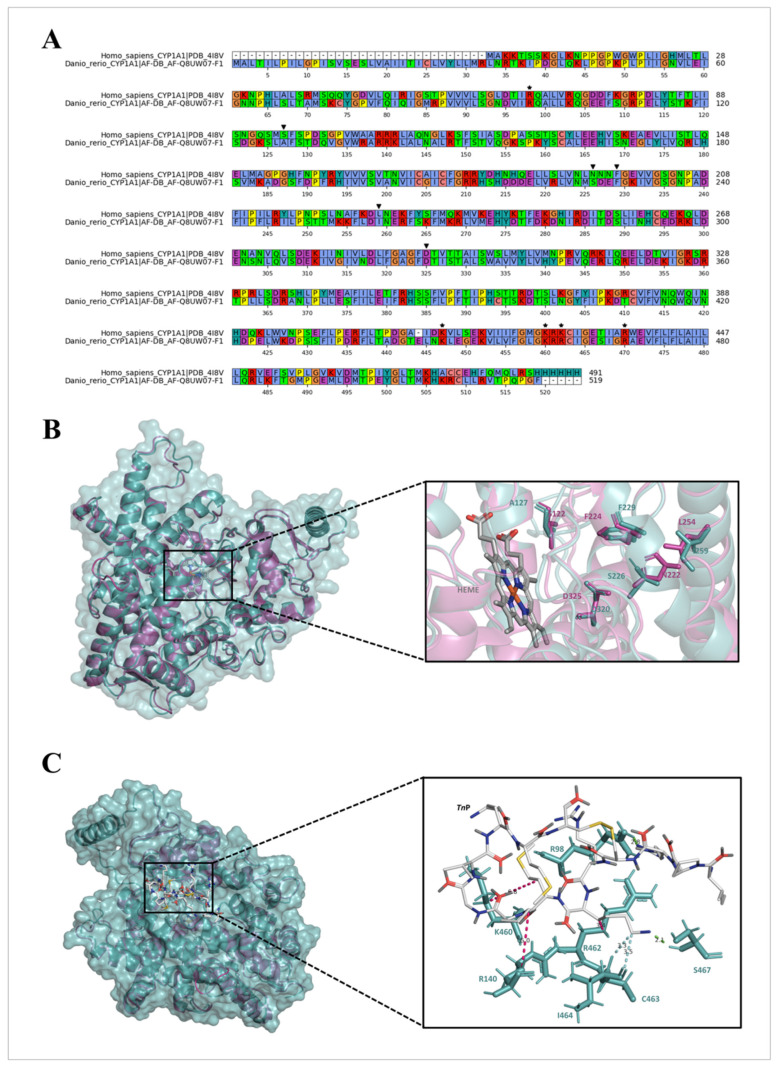
Structure comparison and molecular docking simulations. (**A**). Primary structure alignment of *Homo sapiens* CYP1A1 (PDB: 4I8V) and *Danio rerio* CYP1A1 (AF-DB: AF-Q8UW07-F1). The numbering below the sequences is in accordance with the *D. rerio* CYP1A1 sequence. The residues involved in the active site are labeled by a downward-pointing triangle (▾), and the *Tn*P-binding residues are indicated by asterisks (*). The residues were colored according to Clustal Omega scheme: blue (hydrophobic—A, I, L, M, F, W, and C), red (positively charged—K and R), magenta (negatively charged—E and D), green (polar—N, Q, S, and T), pink (cysteine—C), orange (glycine—G), yellow (proline—P), and cyan (aromatic—H and Y). No present amino acids are indicated by a hyphen (-). (**B**). Tertiary structure comparison between human (purple) and zebrafish (blue) CYP1A1 proteins. On the right side, the active site residues and the heme group are shown in sticks, with the carbon, hydrogen, nitrogen, and oxygen atoms in gray, white, blue, and red, respectively. (**C**). Molecular docking of *Tn*P on *H. sapiens* and *D. rerio* CYP1A1 proteins. The ligand interactions are exhibited on the right side. The peptide is shown in lines with the carbon, hydrogen, nitrogen, and oxygen atoms in gray, white, blue, and red, respectively. The conventional hydrogen bonds, carbon hydrogen bonds, and hydrophobic interactions are shown in green, blue, and pink, respectively.

To further investigate if CYP1 inhibition may play a role in the interaction between the *Tn*P and AHR, we subsequently performed in silico docking analysis of *Tn*P and CYP1A1. The *Homo sapiens* and *D. rerio* CYP1A(1) proteins share 57% identity in the primary structure and have numerous conserved substitutions (Figure 8A). The proteins are highly similar in the tertiary structure, with a root-mean-square deviation (RMSD) equal to 0.72 Å. Interestingly, despite some modifications, both proteins retain the amino acid residues involved in the active site (Figure 8B). Endorsing the *Tn*P inhibition of CYP1A1 activity, we identified the binding sites of *Tn*P on the target. The molecular docking simulations showed that *Tn*P interacts with both human and zebrafish enzymes (Figure 8C), forming hydrogen bonds with Arg^98^, Cys^463^, and Ser^467^, and hydrophobic interactions with Arg^140^, Lys^460^, Arg^462^, and Ile^464^ (Figure 8C).

Moreover, the molecular docking simulations revealed that *Tn*P interacts at the same site in both proteins. Interestingly, the *Tn*P binding site has been recognized as an allosteric site of human CYP1A1, and *Tn*P interacted with allosteric site residues through hydrogen bonds with Arg^98^ (Arg^93^), Arg^462^ (Lys^456^), and Lys^460^ (Lys^454^) [41]. In this site, the heme group is closest to the surface and interacts with the cytochrome P450 oxidoreductase [42,43,44]. Our findings suggest that the interaction with the CYP1A1 allosteric binding site may disrupt the function and inhibit the catalytic activity. Moreover, allosteric binding could induce conformational changes in the active site or cause interferences between CYP1A1 and other proteins [45,46].

## 3. Materials and Methods

### 3.1. Reagents and Chemicals

The peptide *Tn*P trifluoroacetate compound (C_63_H_114_N_22_O_13_S_4_, 97.3% purity, IPR*CRKMPGVKM*C-NH2, MW 1516.00, pI 10.63) synthesized in the solid phase was purchased from GenScripts (#P13821401; Piscataway, NJ, USA); 7-ethoxyresorufin, β-Nicotinamide adenine dinucleotide 2′-phosphate (NADPH, N7505), human recombinant cytochrome P4501A1 with P450 reductase (C3735), Dimethyl Sulfoxide (DMSO), and 3′-methoxy-4′-nitroflavone (MNF) were purchased from Sigma-Aldrich/Merck KGaA (Darmstadt, Germany), and 6-Formylindolo[3,2-b]carbazole (FICZ) was purchased from Chemtronica AB, Sollentuna, Sweden. Morpholinos were obtained from Gene Tools (Philomath, OR, USA).

### 3.2. CYP1A1 Activity In Vitro Inhibition Assay by TnP

The ethoxyresorufin deethylase (EROD) assay was performed using recombinant human CYP1A1 to determine *Tn*P-mediated inhibition of CYP1A1 enzyme activity according to Wincent et al. [47], with minor changes. In brief, 2.5 nM CYP1A1 was pre-incubated with different concentrations of *Tn*P in Tris-HCl buffer (0.1 M, pH 7.4) with EDTA (1 mM) at 37 °C for 3 min followed by the addition of ethoxyresorufin (0.1 μM) and NADPH (0.4 mM). Formation of resorufin was quantified in triplicates using a Tecan Infinite F200 multiwell plate reader (Tecan Group Ltd., Männedorf, Switzerland) with the excitation/emission wavelengths of 530/590 nm every 4 s for 2 min. The enzyme activity was determined by comparing the rate of resorufin formation compared to the vehicle control DMSO. The *Tn*P concentration causing 50% inhibition of EROD activity (IC50) was estimated by nonlinear dose–response inhibition analysis using GraphPad Prism 9.1 (GraphPad Software, San Diego, CA, USA).

### 3.3. Zebrafish Maintenance and Cyp1a Reporter Experiments

Fertilized eggs of zebrafish wild-type AB strain and the transgenic strain Tg(cyp1a:EGFP), obtained from the China Zebrafish Resource Center, Institute of Hydrobiology, Chinese Academy of Sciences [48], were provided by the Karolinska Institutet Zebrafish Core Facility (Solna, Sweden) and kept at the Zebrafish Facility of the Institute of Environmental Medicine (IMM) at 28 °C in a 1× E3 embryo medium (5 mM NaCl, 0.17 mM KCl, 0.33 mM CaCl, and 0.33 mM MgSO_4_ dissolved in MilliQ water, pH 7.0). For manipulation and imaging, embryos were anesthetized with tricaine methanesulfonate (0.02%, MS-222, Sigma-Aldrich, St. Louis, MO, USA). Experimental procedures were conducted using embryos and larvae up to 120 h post-fertilization (hpf). According to the EU Directive 2010/63/EU on the protection of animals used for scientific reasons, the early life stages of fish are not designated as protected and, hence, do not fall into the regulatory frameworks dealing with animal experimentation. Nevertheless, the experimentation followed ethical principles and respected strict animal welfare rules.

At 1 dpf, groups of 30 embryos (in 30 mL of 1× E3 medium) per replicate were exposed to 10 nM FICZ [28], 66 and 199.1 μM *Tn*P, and their binary mixture or solvent control (0.01% DMSO (*v*/*v*)) for up to 120 hpf in glass Petri dishes. All given concentrations are nominal concentrations. AB wild-type embryos were concomitantly exposed to FICZ and used as a control for autofluorescence (which was undetected).

### 3.4. Quantitative Real-Time PCR

The real-time PCR (qPCR) was performed to assess gene expression using the Power SYBR™ Green PCR Master Mix 1X (Applied Biosystems; Cat. no. 4367659) according to the manufacturer’s protocol. The primer sequences used were as follows: *Cyp1a*—F: GCATTACGATACGTTCGATAAGGAC, R: GCTCCGAATAGGTCATTGACGAT; and ribosomal protein L13 (*L13*) as reference—F: GCTAAGGACGGAGTGAACAAC, R: GCACTCTCTTCTGCCAGTC [28]. The reaction was run using the Applied Biosystems QuantStudio 5 Real-Time PCR System (Applied Biosystems, SK, Gothenburg, Sweden) for one cycle at 95 °C for 10 min (hold stage), 40 cycles at 95 °C for 15 s (denaturation), and 60 °C for 1 min (elongation), followed by the conditions necessary for calculating the melting curve, i.e., 15 s at 95 °C, 1 min at 60 °C, and 1 s at 95 °C for dissociation. Relative gene expression was calculated based on the 2^−ΔΔCt^ method.

### 3.5. Experimental Settings for Transcriptome Analysis

#### 3.5.1. *Ahr2* Knockdown, *Tn*P Treatment, and Inflammation Model

Fertilized eggs at the 1–4 cell stage were microinjected either with morpholino-modified oligonucleotides targeting the transcriptional start site of *Ahr2* (Ahr2-MO; 5-TGTACCGATACCCGCCGACATGGTT-3) or standard control morpholino (Ctrl-MO; 5-CCTCTTACCTCAGTTACAATTTATA-3), as described in Wincent et al. [28]. Herein, the *Ahr2*-knockdown embryos are referred to as KD, while their controls are called wild-type (WT). Briefly, the morpholinos were diluted in sterile-filtered deionized water to 0.15 mM. An Eppendorf FemtoJet 4x Microinjector with a fine glass needle was used to inject 2–4 nL of fluorescein-tagged morpholino solution into the yolk of the embryos. The embryos were screened at 6–8 h post-injection (hpi) by fluorescence microscopy to verify successful incorporation and any damaged embryos or those not displaying homogeneous fluorescence were removed.

The zebrafish embryos were raised until 48 hpf when the prophylactic treatment with the 5 or 100 mM *Tn*P stock solution was injected (2–4 nL) in the yolk of manually dechorionated embryos or ultra-pure water as control. Based on the estimated embryo volume of 312 nL/embryo at this time point [49], and assuming a homogeneous distribution, the internal concentration directly after injection (0 hpi, 48 hpf) corresponded to 47.6 and 952.4 µM for the 5 and 100 mM *Tn*P injections, respectively. At 24 hpi (72 hpf), injury-associated inflammation was induced via tail fin amputation [17]. After anesthesia with 0.02% tricaine, the larvae were aligned in glass Petri dishes, and the tip of the tail fin was removed using a scalpel under a stereomicroscope, ensuring a clean and precise amputation. Zebrafish larvae were thereafter placed in a recovery dish for 2 h post-amputation (hpa) before sampling (pool of 10 larvae per group in quadruplicate) in RNAlater Stabilization Solution (Invitrogen, Carlsbad, CA, USA). The RNA preparation and purification were performed using the RNeasy Plus Universal Mini Kit (Qiagen, Hilden, Germany; Cat. No. 73404) following the manufacturer’s instructions.

#### 3.5.2. RNA Quantification and Library Preparation

RNA concentration was estimated using a NanoDrop Spectrophotometer (Thermo Scientific, MA, USA), and the RNA integrity was further assessed using the RNA Nano 6000 Assay Kit of the Bioanalyzer 2100 system (Agilent Technologies, CA, USA) prior to library preparation.

Briefly, mRNA was purified from total RNA using poly-T oligo-attached magnetic beads. Fragmentation was performed using divalent cations under elevated temperature in the First Strand Synthesis Reaction Buffer (5×). First-strand cDNA was synthesized using a random hexamer primer and M-MuLV Reverse Transcriptase (RNase H-). Second-strand cDNA synthesis was performed using DNA polymerase I and RNase H. Remaining overhangs were converted into blunt ends via exonuclease/polymerase activities. After adenylation of 3′ ends of DNA fragments, adaptors with hairpin loop structures were ligated to prepare for hybridization. In order to select cDNA fragments of preferentially 370–420 base pairs (bp) in length, the library fragments were purified with the AMPure XP system (Beckman Coulter, Beverly, CA, USA). Then, PCR was performed with Phusion High-Fidelity DNA polymerase, Universal PCR primers, and Index (X) Primer. Finally, PCR products were purified (AMPure XP system), and library quality was assessed using the Agilent Bioanalyzer 2100 system (Santa Clara, CA, USA).

#### 3.5.3. Clustering and Sequencing

The index-coded samples were clustered on a cBot Cluster Generation System using TruSeq PE Cluster Kit v3-cBot-HS (Illumina, San Diego, CA, USA) according to the manufacturer’s instructions. After cluster generation, the library preparation was sequenced on a state-of-the-art Illumina NovaSeq platform, and 150 bp paired-end reads were generated; a short-reads sequencing strategy was utilized.

### 3.6. Data Analysis

#### 3.6.1. Quality Control and Trimming

Raw data (raw reads) were first evaluated for quality control (QC) and trimmed. This step obtained clean data (clean reads) by removing reads containing adapters, reads containing poly-N, and low-quality reads from raw data. Both steps were performed according to the PreProcSEQ pipeline (https://github.com/resendejss/PreProcSEQ, accessed on 15 May 2023). The FastQC (https://www.bioinformatics.babraham.ac.uk/projects/fastqc/) and MultiQC [50] tools were used in the QC. The Trimmomatic tool [51] was used in the trimming step. All the downstream analyses were based on clean data with high quality (Supplementary Figure S1).

#### 3.6.2. Reads Mapping, Quantification of Mapped Transcripts, Gene Expression Matrix, and Transcript Annotation

The reads were mapped to the *Danio rerio* reference transcriptome provided by Ensembl GRCz11 V.109, and the transcripts were quantified with the Salmon tool, version 0.8.2 [52]. Then, the gene expression matrix was built with the quantified transcripts using the R/Bioconductor Tximeta package, version 1.0.3 [53]. We used the ensemble packages [54] org.Dr.eg.db [55] to annotate the quantified transcripts according to V.109 of the reference ensemble used for quantification.

#### 3.6.3. Reads Grouping

The similarity between the samples was evaluated to verify if the replicates were grouped accordingly. The R dist function was used to calculate the Euclidean distance between the samples, and the PoissonDistance function implemented in the PoiClaClu package [56] to calculate the Poisson distance. As input, the VST-transformed counts (variance stabilizing transformation) were used. The distances on a heatmap were visualized using the pheatmap function from the pheatmap package (https://rdrr.io/cran/PoiClaClu/) (Supplementary Figure S2). In addition, principal component analysis (PCA) was performed with the R pcaExplorer function of the R/Bioconductor package pcaExplorer [57] with an R object of the DESeqDataSet class as input to highlight the differences between groups.

#### 3.6.4. Differential Expression and Gene Ontology Enrichment Analysis

For differential expression (DE) analysis, the counts from the filtered gene expression matrix for protein-coding genes were used. Then, genes that had a sum of all samples and replicates less than 10 were removed. The comparisons assessed here corresponded to (1) control versus injury-induced group (inflamed); (2) injury-induced group versus injury-induced group pre-treated with 5 mM *Tn*P (5 mM *Tn*P/inflamed); and (3) injury-induced group versus injury-induced group pre-treated with 100 mM *Tn*P (100 mM *Tn*P/inflamed). All the comparison groups involving induced inflammation and *Tn*P treatment and their multiple comparisons underwent DE analysis using the R/Bioconductor DESeq2 package [58]. *p*-values were calculated using the Wald test and adjusted using Benjamini and Hochberg’s approach for controlling the false discovery rate, and genes with an adjusted *p*-value ≤ 0.05 were assigned as differentially expressed genes (DEGs); additionally, we used the log_2_ fold change ±0.5 as a threshold for significance (DEGs = padj < 0.05, log_2_FC ± 0.5). Genes obtained from the DE lists were subjected to enrichment analysis. The R/Bioconductor clusterProfiler [59] and org.Dr.eg.db [55] packages were used to assess the Gene Ontology (GO) enrichment analysis. GO terms with adjusted *p*-values less than 0.05 were considered significantly enriched. We used the clusterProfiler R package to test the statistical enrichment of differential expression genes in Kyoto Encyclopedia of Genes and Genomes (KEGG) pathways (http://www.genome.jp/kegg/).

### 3.7. In Silico Analysis

To build up with the CYP1A1 inhibition assay, the *Tn*P binding sites were investigated. Firstly, the *Tn*P tertiary structure was modeled using PEPstrMOD [60]. Next, we analyzed the similarity between the *Homo sapiens* CYP1A1 (PDB: 4I8V) [61] and *Danio rerio* CYP1A1 (AlphaFold-DB: AF-Q8UW07-F1) [62] in the primary structure through sequence alignment using Clustal Omega v.1.4.2 [63,64] and visualized with the Python (https://www.python.org/) package pyMSAviz v.1.2 [65]. Moreover, we evaluated the similarity between the tertiary structures by measuring the root-mean-square deviation (RMSD) using PyMOL v. 2.5.7 (Schrödinger, Inc., New York City, NY, USA). Then, we performed molecular docking simulations using GOLD v. 2023.3.1 [66]. The remaining ions and water molecules were removed from the crystal structure before the analysis in the target preparation. In addition, hydrogen atoms were added to correct the protonation states of the amino acid residues. By the coordinates of the heme group, a 15 Å grid was determined for atom selection and detection of the presence of solvent-accessible surfaces. Hydrogen bond donors and acceptors were treated as accessible to solvent. The Piecewise Linear Potential (CHEMPLP) fitness function was used to rank molecular docking solutions. The CHEMPLP function models the stereochemical complementarity of the protein and the ligand in terms of angle-dependent hydrogen bonding and torsional potential [67]. The docking simulation was set to 10 interactions, and the efficiency search was determined at 200% with the highest number of operations to increase the accuracy in mapping the complementarity of the ligand and the target proteins. The protein and ligand binding and interactions were visualized using Discovery Studio Visualizer v. 21.1.0.20298 [68] and PyMOL v.2.5.7.

### 3.8. Statistical Analysis

Statistical analysis was performed using GraphPad Prism (GraphPad Software, v6.02, CA, USA). Comparisons between group treatments over several time points were tested using two-way ANOVA, followed by Dunnett’s multiple comparisons test with a significance level of *p* > 0.05. Meanwhile, differences in gene expression assessed by qPCR were verified using the delta–delta Ct method, also known as the 2^−∆∆Ct^ method, to calculate the relative fold gene expression. In this case, log_2_ of 2^−∆∆Ct^ was compared by an ordinary one-way ANOVA followed by Tukey’s multiple comparisons test. The specificities of transcriptomic analysis are described in their corresponding topics.

## 4. Conclusions

The AHR has long been implicated in regulating a battery of genes necessary for metabolizing endogenous compounds and xenobiotics. In addition, its function as a transcription factor places it in many physiological and pathological processes, particularly in the immune and inflammatory responses, stem cell maintenance, cellular differentiation, cell survival, and tumorigenesis. Since the interface between physiological, adaptive, and toxicological action elicited by AHR-mediated responses is still elusive, in-depth research is required to evaluate its toxicological and physiological features. Considering the crucial role of CYP1 in regulating physiological AHR functions, it is vital to evaluate the role of this feedback mechanism also when assessing xenobiotic toxicity and when developing therapeutic strategies targeting AHR.

In the context that CYP1 inhibition may ameliorate inflammation, reduce oxidative stress, and alter AHR activity, it could be a good fit for *Tn*P therapy, as demonstrated by its modulation of inflammatory signaling through the AHR-CYP axis. One possibility is combinatorial drug therapy (i.e., *Tn*P and other drugs), which could be used to enhance the therapeutic effects of *Tn*P through AHR-dependent and -independent mechanisms. Additionally, CYP inhibition can prolong the half-life of drugs, which may be advantageous in cases where steady and sustained drug levels are required to manage a chronic condition.

Predicting drug–drug interactions is significant in drug development in the pharmaceutical industry and regulatory agencies. Overall, the results presented here highlight that the zebrafish injury-induced inflammation model can provide valuable insights into molecular tuning by drugs on the acute immune response and stresses that *Tn*P modulates the molecular profiles induced by inflammation through both AHR-dependent and -independent mechanisms.

## Data Availability

Data is contained within the article and Supplementary Material.

## Supplementary Materials

The following supporting information can be downloaded at https://www.mdpi.com/article/10.3390/ph17091155/s1, Figure S1: Quality control analysis of experimental samples submitted to RNA sequencing before (A, C, E) and after data filtering (B, D, F); Figure S2: Distance matrix- Poisson (A) and Euclidean (B) method/distribution to describe overall RNAseq data; Figure S3: Transcriptional response profile modulated by the *Tn*P treatment in a zebrafish inflammation model considering the presence, wild-type (WT; A-C) and knockdown (KD; D-F) of AHR; Figure S4: Venn diagram of the differentially expressed genes (DEG) among wild-type (WT; top) and *Ahr2*-knockdown (KD, bottom) groups; Table S1: Range of *Tn*P concentrations and inhibitory effect on the recombinant CYP1A1 enzyme (Cyp1a inhibition assay); Table S2. Differential Expression Analysis; Table S3. GO Enrichment Analysis.
