# Supplementary material for: TnP and AHR-CYP1A1 Signaling Crosstalk in an Injury-Induced Zebrafish Inflammation Model
Source: Pharmaceuticals (Basel). 2024 Aug 31;17(9):1155. doi: 10.3390/ph17091155 (PMC11435205; doi:10.3390/ph17091155)
Supplement: Supplementary file 1 [file pharmaceuticals-17-01155-s001.zip › pharmaceuticals-3169293-supplementary Figures.pdf]

SUPPLEMENTARY MATERIAL

FIGURES

Supplementary Figure S1. Quality control analysis

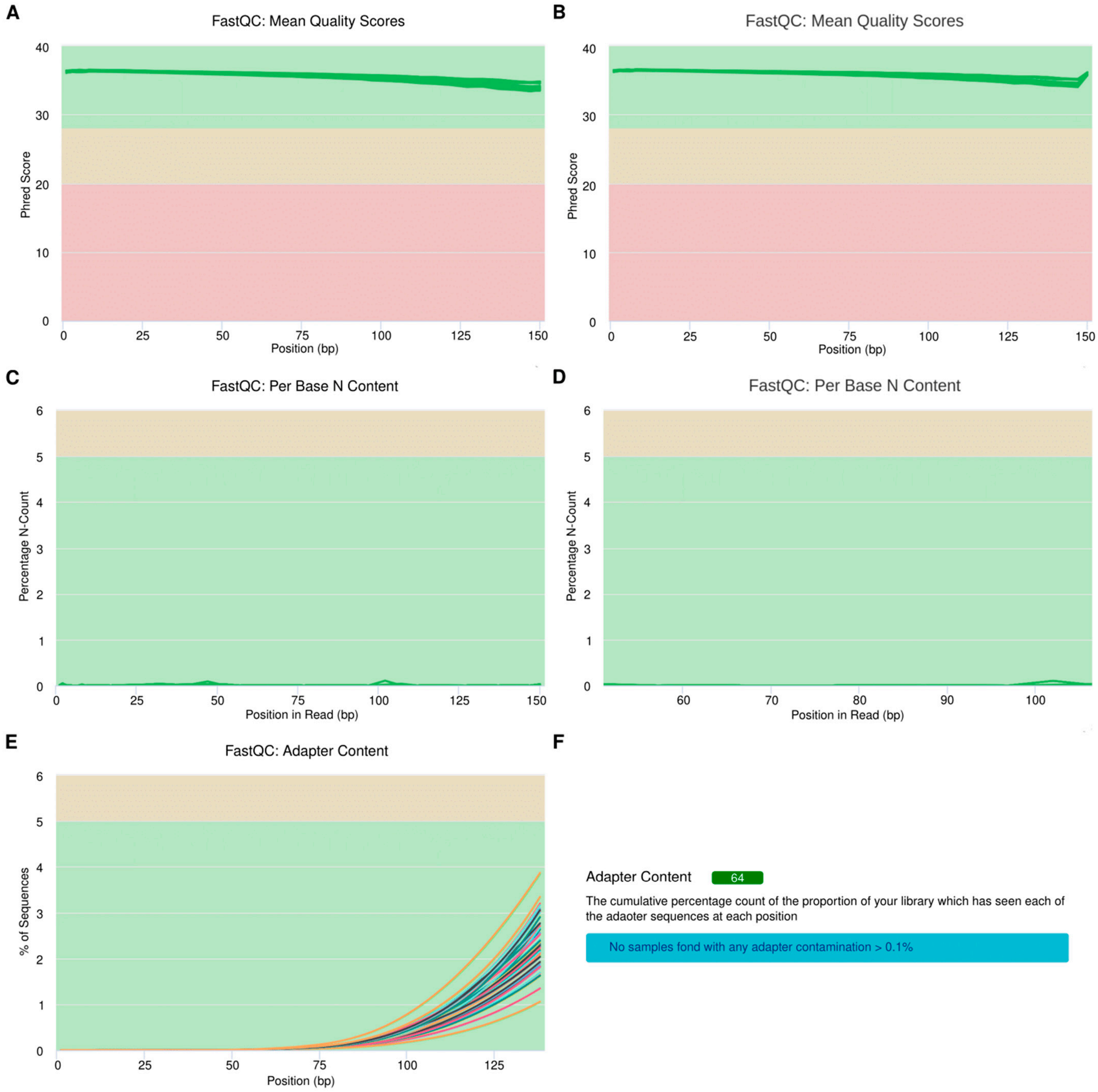

Figure S1: Quality control analysis of experimental samples submitted to RNA sequencing before (A, C, E) and after data filtering (B, D, F). The sequencing reads/raw reads often contain low-quality reads or reads with adapters, which will affect the quality of downstream analysis. To avoid this, it is necessary to filter the raw reads and obtain the clean reads. Those metrics are calculates as follow: Adapter related= (reads containing adapter) / (total raw reads); Containing N= (reads with more than 10% N) / (total raw reads); Low quality= (reads of low quality) / (total raw reads); Clean reads= (clean reads) / (total raw reads). On the other hand, raw reads filtering workflow is performed as follows: i) Remove reads with adapter contamination; ii) Remove reads when uncertain nucleotides constitute more than 10 per cent of either read (N > 10%); and iii) Remove reads when low-quality nucleotides (Base Quality less than 5) constitute more than 50 per cent of the read. The adapter sequences were: P5 Adapter - P5-AATGATACGGCGACCACCGAGA (5′-3′) and P5′-TTACTATGCCGCTGGTGGCTCT (3′-5′); P7 Adapter - CGTATGCCGTCTTCTGCTTG-P7′ (5′-3′) and GCATACGGCAGAAGACGAAC-P7 (3′-5′).

Supplementary Figure S2. Samples distribution matrix

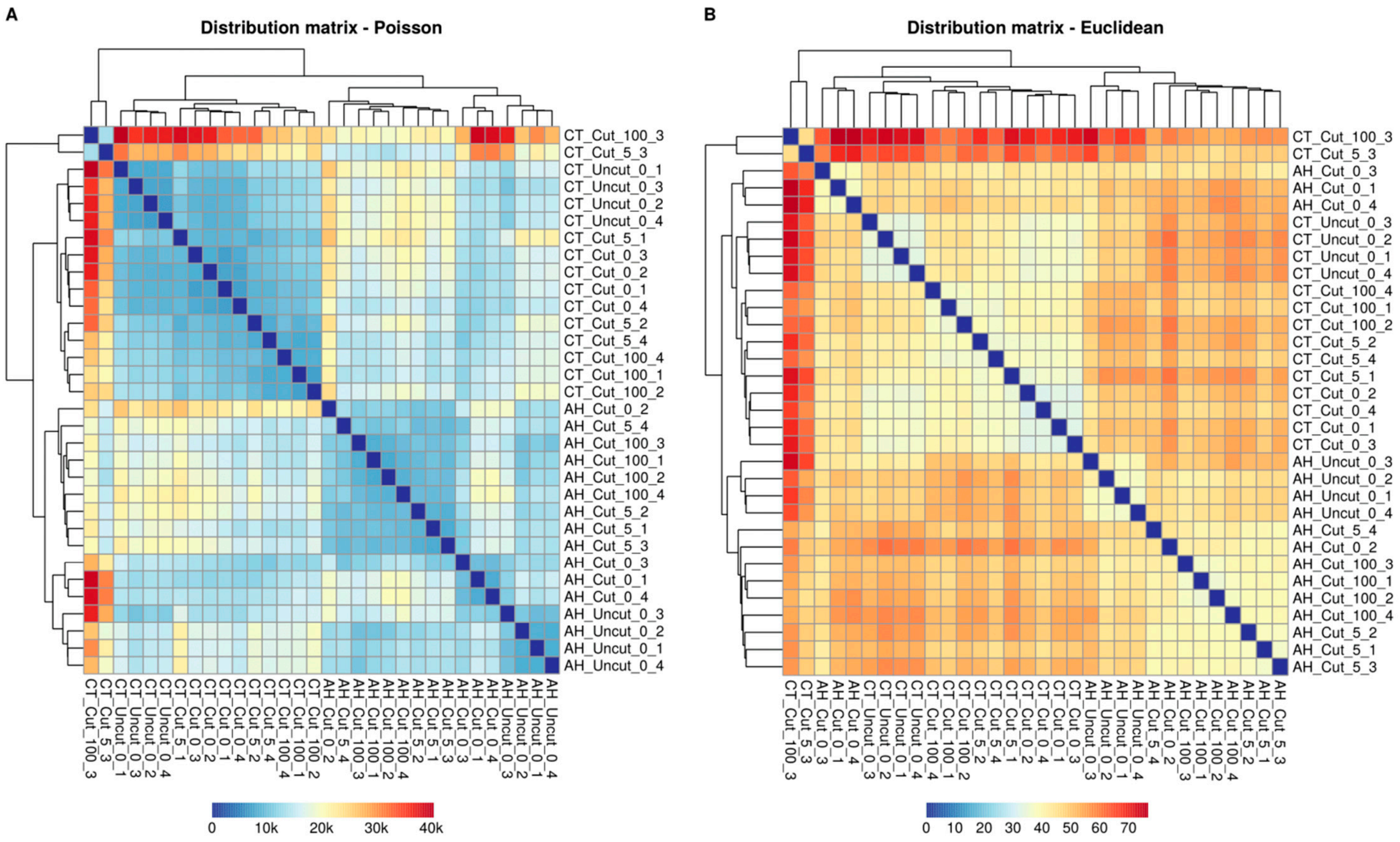

Figure S2: Distance matrix- Poisson (**A**) and Euclidean (**B**) method/distribution to describe overall RNAseq data. <u>Sample coding</u>: CT – wild-type zebrafish (control); AH – *ahr2*-knockdown groups; Cut – embryos subjected to wound-induced tail fin amputation; Uncut – control groups not inflamed; 100 – prophylactic treatment with 100 mM *Tn*P; 5 – prophylactic treatment with 5 mM *Tn*P; 0 – control groups not treated with *Tn*P. Each experimental group was sequenced in quadruplicate (1–4).

Supplementary Figure S3. Volcano plots from differential expression analysis [DEG (padj<0.05, $\log_2$FC±0.5)]

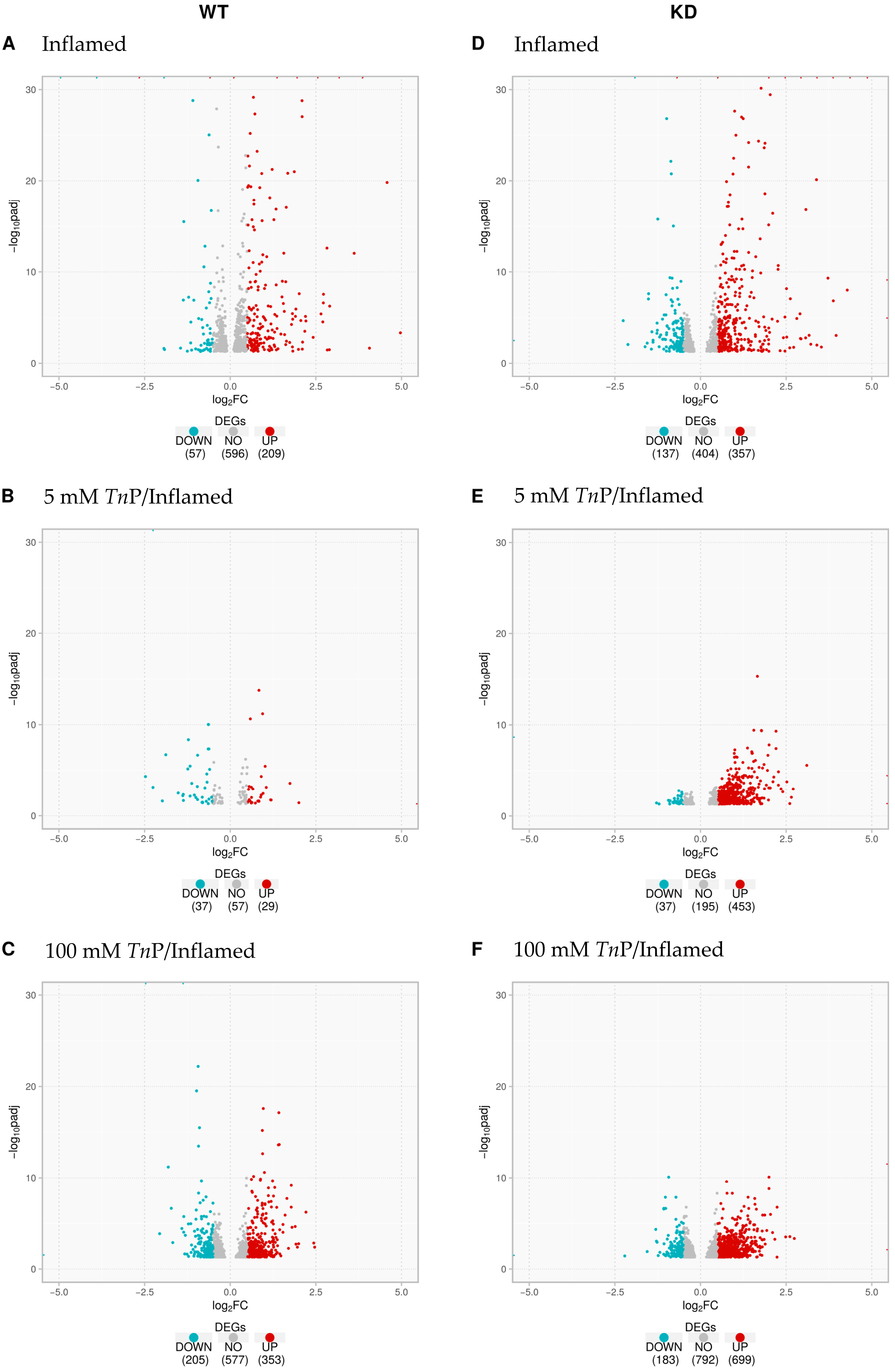

Figure S3: Transcriptional response profile modulated by the *Tn*P treatment in a zebrafish inflammation model considering the presence, wild-type (WT; A-C) and knockdown (KD; D-F) of AHR. **A**) WT Inflamed;

B) WT 5 mM *Tn*P/Inflamed; **C**) WT 100 mM *Tn*P/Inflamed; **D**) KD Inflamed; **E**) KD 5 mM *Tn*P/Inflamed; **F**) KD 100 mM *Tn*P/Inflamed. Level of significance set to padj <0.05 and log_2FC±0.5.

Supplementary Figure S4. Venn diagrams with DEG number among conditions/treatments within genotypes

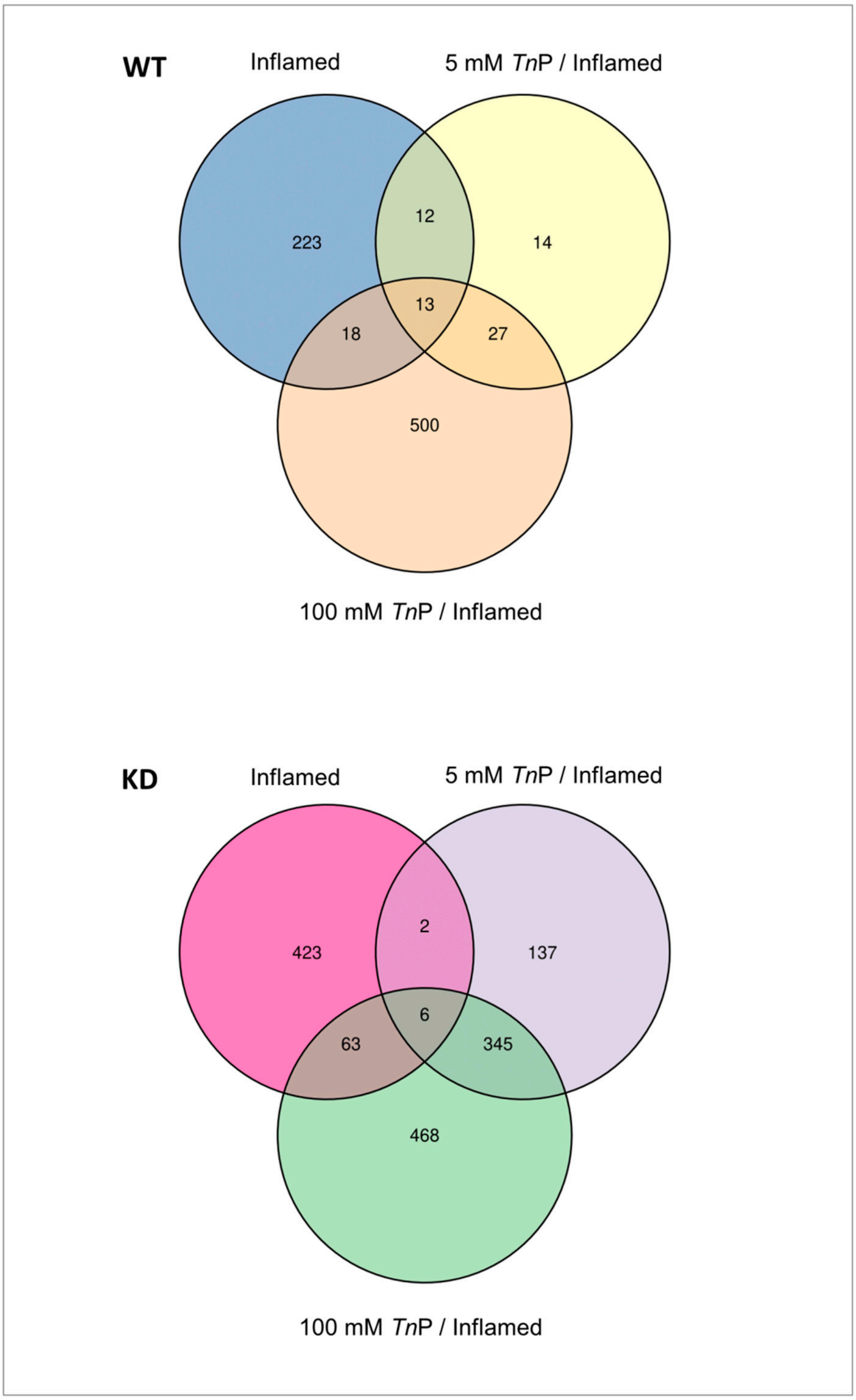

Figure S4: Venn diagram of the differentially expressed genes (DEG) among wild-type (WT; top) and *Ahr2*-knockdown (KD, bottom) groups. The number in each circle represents the amount of DEGs between the different comparisons (Inflamed *vs*. 5 mM *Tn*P/Inflamed *vs*. 100 mM *Tn*P/Inflamed. The overlapping number stands for the mutual DEGs between the different comparisons and the non-overlapping numbers specify the genes unique to each condition.
