# Supplementary material for: TnP and AHR-CYP1A1 Signaling Crosstalk in an Injury-Induced Zebrafish Inflammation Model
Source: Pharmaceuticals (Basel). 2024 Aug 31;17(9):1155. doi: 10.3390/ph17091155 (PMC11435205; doi:10.3390/ph17091155)
Supplement: Supplementary file 1 [file pharmaceuticals-17-01155-s001.zip › pharmaceuticals-3169293-supplementary Table S1.pdf]

Supplementary Table S1: Range of *Tn*P concentrations and inhibitory effect on the recombinant CYP1A1 enzyme (Cyp1a inhibition assay). Percentage of the enzyme activity of each replicate; *Tn*P in µM. All the concentration were read at least in triplicate. Dimethyl sulfoxide – DMSO was tested asnegative control, and 3′-methoxy-4′-nitroflavone – MNF as positive control.

Treatment	Concentration (µM)	Activity (%) I	Activity (%) II	Activity (%) III	Activity (%) IV	Activity (%) V	Activity (%) VI	Activity (%) VII	Activity (%) VIII	Activity (%) IX
DMSO	0.1%	100								
*Tn*P	0.0825	94,49	94,02	94,49	109,70	109,70	110,14			
	0.33	99,96	100,71	93,94	98,86	97,08	96,01			
	6.6	106,92	108,52	107,84						
	0.825	102,97	102,63	102,63	106,75	107,28	107,28			
	1	81,89	81,84	81,84						
	2	93,85	93,85							
	8.25	93,29	93,69	91,40						
	16.5	94,47	93,09	93,09						
	33	87,06	85,81	84,78						
	66	70,14	72,68	70,14	80,17	76,49	76,49	82,23	81,16	80,03
	82.5	72,45	69,67	69,67	85,56	85,56	85,09			
	165	48,87	47,76	47,76						
	330	36,56	36,70	37,60						
	660	22,83	22,35	22,40						
MNF	2.5	1,54	1,54	1,71	1,67	1,31	1,70	0,51	0,51	0,51
